# The Isolation Hospital Movement: An Authoritative Institutional Textbook

**Published:** 1914-05-30

**Authors:** 


### THE ISOLATION HOSPITAL MOVEMENT.

### An Authoritative Institutional Textbook.

The lamented death of Dr. H. Franklin Parsons took place when the proofs of an important work from his practised pen were being passed through the press. This was a textbook dealing with isolation hospitals, which he had undertaken for the Cambridge Public Health Series.* On this particular side of institutional construction, management, and administration, the author was one of the very first authorities; for during his long service under the Local Government Board he had frequently been employed in reporting on schemes, designs, and proposals of all sorts in connection with the treatment by local authorities of infective diseases. To members of local authorities, to medical officers of health, and to superintendents and architects of isolation hospitals his work makes a special appeal which must be irresistible. We know of no monograph at once so complete, so up to date, so broad minded, and so well written as this posthumous publication; while congratulating the institutional world on the fact that the author completed it, we can only regret that he did not live to receive the appreciation which it is certain to call forth from those for whom it was written. Among them we may count particularly our readers, since his signed articles in The Hospital were fairly frequent of late years, and always commanded respect from their knowledge and attention from their style, which was lucid, direct, and engaging.

Vast as have been the strides made in the institutional treatment of infective diseases during the last two generations, there are still sundry vexed topics upon which agreement among the experts has not been reached. Dr. Parsons takes a very fair line about such topics: he does not hesitate to give his own opinion, but he also makes a successful effort to state fairly the opposing position when there is one. In a good many cases he adopts a middle course, believing, as he himself says, that the truth will probably be found somewhere between the extreme views, or perhaps in some altogether different and unexpected direction. Such controversial subjects, for instance, are the utility of hospital isolation of scarlet fever (he pronounces no opinion of his own about the Milne treatment, and his quotations from literature include both favourable and unfavourable reports); the aerial convection of infection from smallpox hospitals; and bed-isolation in relation to the prevention of cross-infection and of "return cases."

### Meaning of Bed Isolation.

He holds that bed isolation, whether by structural separation or by aseptic methods of nursing, affords the most likely way out of the difficulties and drawbacks attendant upon the hospital treatment of infectious diseases. There is reason to believe, he says, that in infectious diseases the primary infection is liable to have accompanying or supervening upon it other secondary septic infections, to which latter the severity of the resulting illness and the occurrence of complications are largely due. These secondary infections may be different even where the primary disease is the same; and the logical deduction from these premises is this, that every patient suffering from an acute infectious disease should be required to be isolated, more or less completely, according to circumstances, not only from healthy susceptible persons and from those suffering from other kinds of infectious disease, but also *from other cases of the same disease.*

With regard to scarlet fever, Dr. Parsons admits that in the present mild phase of this disease many of the patients now removed to isolation hospitals would do just as well at home; and that there would be much saving to the rates and little detriment to the public health if the removal of scarlet fever cases were limited to exceptional cases of special urgency. But he states that hospital isolation has become such a convenience that any such limitation would give rise to grave dissatisfaction, especially among the working classes.

### The Old Controversy.

How greatly public (and medical) opinion has changed over the isolation of infectious fevers is well shown by an extract, which the author quotes, from the writings of the great Murchison, who in 1873 quoted a contemporary fulmination that fever hospitals and fever wards were "a crime against humanity and a disgrace to the age in which we live." Needless to say, Murchison himself did not uphold this dictum, but his views were strongly opposed by Dr. J. S. Bristowe and Mr. T. Holmes in their Report on the Hospitals of the United Kingdom to the Privy Council. Space does not permit us to follow Dr. Parsons into his careful account of the history and development of the isolation hospital movement, to which a good bibliography is appended.

Of greater importance, recognised by the larger space allotted to them, are such matters as the selection of sites for isolation hospitals, the design of hospitals of all sizes, and the constructional details of ward blocks. Into all these Dr. Parsons enters with enthusiasm and expert knowledge; no detail is petty enough to escape his eye—lighting, drainage, sanitation, bathrooms, hot-water supply, ventilation, warming, floors, ceilings, walls, all receive due consideration. Then follows a useful section on hospitals of more or less perishable construction, such as tents and portable structures of various kinds. Smallpox hospitals, as being in many ways *sui generis*, receive a special chapter; as also do port sanitary hospitals. The removal of patients to hospital is then reviewed, and the

* *Isolation Hospitals.* By H. Franklin Parsons, M.D., D.P.H. (Cambridge University Press. 1914. Pp. 275. Price 12s. 6d. net.)

various types of vehicle are compared. Incidentally the author notes that the Act which allows local authorities (outside London) to pay the cost of conveying patients to their isolation hospitals does not empower them to convey the patients back from hospitals to their homes; this is only a small point, but it serves to show the thoroughness of the book. The hospital system of the Metropolitan Asylums Board, the cost of isolation hospitals, and sanatoria for tuberculosis are also dealt with; and a chapter full of plans of actual isolation hospitals in different parts of the kingdom concludes the book. The plans and designs, freely illustrated, together with the bibliographies, are uniformly good; these and the admirable setting of the book by the University Press combine to increase its very great value to the institutional world.

